# *Notes from the Field:* Amphetamine Use Among Workers with Severe Hyperthermia — Eight States, 2010–2019

**DOI:** 10.15585/mmwr.mm6930a5

**Published:** 2020-07-31

**Authors:** Andrew S. Karasick, Richard J. Thomas, Dawn L. Cannon, Kathleen M. Fagan, Patricia A. Bray, Michael J. Hodgson, Aaron W. Tustin

**Affiliations:** ^1^Directorate of Technical Support and Emergency Management, Occupational Safety and Health Administration, U.S. Department of Labor; ^2^Environmental and Occupational Health Sciences Institute, Rutgers University, Piscataway, New Jersey.

Workers can develop hyperthermia when core body temperature rises because of heat stress (environmental heat plus metabolic heat from physical activity) (*1*). Amphetamines are central nervous system stimulants that can induce hyperthermia independently or in combination with other risk factors (*2*). During 2010–2016, the Directorate of Technical Support and Emergency Management’s Office of Occupational Medicine and Nursing (OOMN), at the Occupational Safety and Health Administration (OSHA), identified three workers with fatal hyperthermia who tested positive for methamphetamine (*3*). To identify additional cases of severe hyperthermia in which workers tested positive for amphetamines, and to support OSHA’s enforcement activities, OOMN reviewed all medical records and investigation materials submitted by other OSHA offices to OOMN during January 1, 2010–August 31, 2019. OSHA field offices obtained the records from employers and health care facilities as part of OSHA’s inspections to enforce occupational safety and health regulations. Confirmed severe hyperthermia was defined as highly elevated body temperature (e.g., core temperature ≥104°F [40°C] or peripheral temperature ≥102°F [38.9°C]) associated with death or serious central nervous system dysfunction (e.g., coma or seizure). For out-of-hospital deaths with no body temperature measurement, suspected severe hyperthermia was defined as a determination by a medical examiner or other responsible postmortem investigator that hyperthermia caused or contributed to the death. The record review identified 111 heat-related illnesses, 46 of which involved severe hyperthermia (38 fatal and eight nonfatal illnesses).

Toxicology results (e.g., urine drug screens or postmortem blood tests) were available in 34 (73.9%) of the 46 cases of severe hyperthermia (including the three previously mentioned methamphetamine cases). Nine (26.5%) of these 34 workers tested positive for an amphetamine-class substance.* All nine were adult males aged 18–47 years (median = 30 years) working in various industrial settings in eight U.S. states^†^ on warm days in summer or late spring (Table). Based on data from the nearest National Weather Service observation stations, the maximum outdoor heat index (a metric that combines temperature and relative humidity into a single number that represents how hot the conditions feel to humans) ranged from 86°F to 107°F (median = 97°F) on the days of the nine incidents.

**TABLE:** Characteristics of nine male workers with severe hyperthermia who tested positive for amphetamines — eight states, 2010–2019

Worker	Age (yrs)	Industry category	Month of event	Maximum outdoor heat index* (°F)	State	Highest measured body temperature (°F)	Legal amphetamine prescription	Outcome	Co-occurring substances^†^
A	47	Construction	August	101	Texas	108.2	No	Survived	Tetrahydrocannabinol
B	18	Landscaping	June	86	Ohio	106.6	Yes	Died	Tetrahydrocannabinol
C	25	Manufacturing	July	105	Texas	109.7	No	Survived	Benzodiazepine, opioid
D	30	Construction	August	97	Rhode Island	110.6	Yes	Died	Tricyclic antidepressant, benzodiazepine, antihistamine
E	32	Waste collection	June	95	Florida	103.0	No	Died	Tetrahydrocannabinol
F	36	Oil and gas extraction	June	97	Oklahoma	109.9	No	Died	None
G	30	Oil and gas extraction	July	95	Kansas	110.0	No	Died	Tetrahydrocannabinol
H	47	Landscaping	August	107	Missouri	Not measured^§^	No	Died	Caffeine
I	26	Construction	July	88	Nebraska	106.3	No	Died	None

* Heat index combines ambient temperature and relative humidity into a single metric that quantifies how hot the conditions feel to humans (https://www.weather.gov/safety/heat-index).

^†^ Excludes medications administered during post-incident resuscitation and treatment efforts.

^§^ This worker died unattended and had rigor mortis when he was found. No vital signs were recorded.

Seven of the nine workers died, and two survived life-threatening illnesses. Peak body temperature ranged from 103°F to 110.6°F (39.4°C to 43.7°C) in eight workers with confirmed severe hyperthermia. In one fatality with no premortem body temperature measurement, the medical examiner suspected that hyperthermia was a significant contributing condition, based upon the circumstances (i.e., death occurred in a hot environment after strenuous activity on a hot day) and lack of anatomic evidence of an alternative cause of death (e.g., myocardial infarction).

According to medical records and medical examiner reports obtained by OSHA, illicit amphetamine use appeared to be present in seven cases; three postmortem blood assays detected methamphetamine, and four qualitative screening tests detected amphetamine or amphetamine analogs in workers without amphetamine prescriptions. One of the latter four workers died of hyperthermia on his first day at a new job, after reportedly receiving a drug from his supervisor. In that case, a coworker later alleged to OSHA that before the shift started, the supervisor had provided pills whose appearance was consistent with those of a prescription amphetamine. Two cases involved legal use of prescription amphetamines to treat attention deficit hyperactivity disorder, and both persons who used legal prescription amphetamines died. Co-occurring substances detected by blood or urine toxicology testing included tetrahydrocannabinol (four patients), benzodiazepines (two), opioids (one), tricyclic antidepressants (one), antihistamines (one), and caffeine (one). Clinicians and investigators determined that these co-occurring substances were not causally related to the hyperthermia outcomes.

This investigation revealed a high prevalence (>25%) of amphetamine use among 34 workers with severe hyperthermia. CDC’s National Institute for Occupational Safety and Health (NIOSH) has found that amphetamines are associated with heat intolerance (*1*), but reports of workplace hyperthermia where amphetamines were detected are limited (*4*). Workers and supervisors should be aware of potential hyperthermia-inducing synergy between amphetamines, physical activity, and environmental heat. Workers should not use illicit amphetamines to maintain alertness or enhance performance, especially when heat stress is present. Prevention of illicit amphetamine use is important, not only to avert hyperthermia but also to prevent other adverse effects. Workers should receive support for overcoming stimulant use disorders.^§^ Clinicians who prescribe amphetamines should consider obtaining an occupational history to facilitate discussions with patients about heat stress safety. Stakeholders should implement comprehensive occupational heat stress controls, such as those recommended by NIOSH (*1*) and OSHA (*5*), to prevent illnesses.

## Footnotes

† Florida, Kansas, Missouri, Nebraska, Ohio, Oklahoma, Rhode Island, and Texas.
